# Effects of Dichlorodiphenyltrichloroethane on the Female Reproductive Tract Leading to Infertility and Cancer: Systematic Search and Review

**DOI:** 10.3390/toxics11090725

**Published:** 2023-08-24

**Authors:** Shermeen Syed, Shandana Qasim, Maheen Ejaz, Sammar, Nimra Khan, Haider Ali, Himasadat Zaker, Eleftheria Hatzidaki, Charalampos Mamoulakis, Aristidis Tsatsakis, Syed Tahir Abbas Shah, Saira Amir

**Affiliations:** 1 Functional Genomics and Proteomics Laboratory, Department of Biosciences, COMSATS University Islamabad, Park Road Chak Shehzad, Islamabad 44000, Pakistan; shermeensyed99@gmail.com (S.S.); shandanaqasim@gmail.com (S.Q.); maheenejaz26@gmail.com (M.E.); sammarmalik903@gmail.com (S.); nimraaakhan88@gmail.com (N.K.); syedtahirabbas@comsats.edu.pk (S.T.A.S.); 2 Cerebral Venous Disorder Lab, University of California, San Francisco, CA 94143, USA; haiderali00pk@gmail.com; 3 Histology and Microscopic Analysis Division, RASTA Specialized Research Institute (RSRI), West Azerbaijan Science and Technology Park (WASTP), Urmia 5756115322, Iran; zakerhima@gmail.com; 4 Department of Neonatology, University General Hospital of Heraklion, Medical School, University of Crete, 71003 Heraklion, Crete, Greece; el.hatzidaki@uoc.gr; 5 Department of Urology, University General Hospital of Heraklion, Medical School, University of Crete, 71003 Heraklion, Crete, Greece; mamoulak@uoc.gr; 6 Toxicology Lab, Department of Medicine, University of Crete, 71003 Heraklion, Crete, Greece; tsatsaka@uoc.gr; 7 Department of Human Ecology and Environmental Hygiene, IM Sechenov First Moscow State Medical University, Moscow 119991, Russia

**Keywords:** dichlorodimethyltrichloroethane, endocrine disruptors, female, infertility, neoplasms, Persistent Organic Pollutants

## Abstract

Persistent Organic Pollutants (POPs) such as dichlorodimethyltrichloroethane (DDT) are present and ubiquitous in the environment due to their resilient nature. DDT is a prevalent endocrine disruptor still found in detectable amounts in organisms and the environment even after its use was banned in the 1970s. Medline and Google Scholar were systematically searched to detect all relevant animal and human studies published in the last 20 years (January 2003 to February 2023) in accordance with the Preferred Reporting Items for Systematic Reviews and Meta-Analyses (PRISMA) statement. In total, 38 studies were included for qualitative synthesis. This systematic search and review indicated that exposure to DDT is associated with female reproductive health issues, such as reduced fecundability; increased risk of preterm/premature deliveries; increased periods of gestation; alterations in the synthesis of crucial reproductive hormones (Progesterone and Oxytocin) through ion imbalances and changes in prostaglandin synthesis, myometrial and stromal hypertrophy, and edema; and variations in uterine contractions through increased uterine wet weight. There was also limited evidence indicating DDT as a carcinogen sufficient to instigate reproductive cancers. However, this review only takes into account the in vitro studies that have established a possible pathway to understand how DDT impacts female infertility and leads to reproductive cancers. Links between the pathways described in various studies have been developed in this review to produce a summarized picture of how one event might lead to another. Additionally, epidemiological studies that specifically targeted the exposure to DDT of females belonging to various ethnicities have been reviewed to develop an overall picture of prevailing female reproductive health concerns in different nations.

## 1. Introduction

Dichlorodiphenyltrichloroethane (DDT) is an organochlorine pesticide (OCP) classified as a Persistent Organic Pollutant (POP) [1]. POPs are a group of organic compounds resistant to degradation, with long half-lives. Their ability to bioaccumulate through different trophic levels, terrestrial and aquatic, and their potential to be transported over large distances by air or water, render them immobilized, particularly due to their hydrophobic nature and through a process known as Long-Range Atmospheric Transport (LRAT). This transport of DDT is also suggested to be directly or indirectly influenced by changes in temperature, humidity, solar radiation, and wind speed. This indicates that climate change also plays a role in its presence in the environment [2]. DDT was initially used to treat insect-borne diseases such as malaria and typhus in humans [3], and later as an agricultural pesticide in the 1940s and 1950s [4]. Due to its adverse environmental and toxicological effects, and its persistence in the environment attributed to its stable structure, the use of DDT in agriculture was banned in the US in the 1970s, in Asia in the 1980s and 1990s, and worldwide in 2001 by the Stockholm Convention on POPs. DDT is still used to control vector-borne diseases in South Africa, Uganda, Ethiopia, and the Kingdom of Eswatini [5]. However, even after its widespread use was discontinued, it has still been detected in toxic amounts in humans and animals. Exposure to DDT occurs through different routes such as inhalation, ingestion, and contact with the skin.

The primary route of DDT entry is consumption of contaminated meat, fish, and crops with high fat content. It can accumulate in breast milk fat, passing to infants through breastfeeding [6]. DDT can also cross the placental barrier [7]. In utero exposure to DDT is linked with reduced birth weight and length, reduced head circumference, preterm birth, and fetal loss by interfering with hormonal balance, receptor–ligand interactions, immunotoxicity, and ion imbalances, which lead to alterations in uterine and myometrial contractions and prostaglandin synthesis, and can result in edema and hypertrophic conditions [8]. Lactational transfer is significantly higher than placental transfer. Effects of DDT include toxicity of the liver and central nervous system, reproductive cancers, and estrogenic, antiandrogenic, and epigenetic effects [9,10]. Exposure to DDT can also have transgenerational effects such as obesity, early onset of puberty, insulin resistance, and testis, ovary, and kidney pathologies in the offspring of exposed organisms [11,12]. Ancestral DDT exposure was found to significantly increase the incidence of polycystic ovaries and the development of uterine infections in future generations in rats [13]. Fetal, neonatal, or pubertal exposure cause impaired reproductive function. DDT and its metabolites can cause a widespread reduction in population size by accumulating in the eggs of birds and reptiles, affecting the eggshell thickness and consequently decreasing reproductive success [14]. It reduces fertility in mice, causing a decline in sperm quality and quantity, and rendering them sterile after exposure [15]. DDT and its metabolites are endocrine-disrupting chemicals (EDCs) which mimic the action of estrogen, disturbing the estrogen receptor (ER) pathways [16]. It also displays anti-androgenic effects in combination with the androgen receptor (AR). DDT induces a disrupter effect in target cells, which involves competing with testosterone to bind to AR, causing receptor-signaling impairment. It also increases estrogen synthesis, implicating hormone production and alterations that increase the probability of somatic and reproductive disorders in later life [16]. An imbalance in the production of testosterone and estrogen due to excessive estrogen production is actively associated with an increased risk of feminization. It can also trigger estrogen-related cancers and cardiovascular disorders [17,18]. Studies show that exposure to DDT can cause adverse health effects in humans and animals by impairing fertility and increasing the risk of developing cancer. Thus, the aim of this work is to provide a comprehensive overview of the available literature to assess the possible impact of DDT and its metabolites on female reproductive health, leading to infertility and cancer. This review only takes into account the in vitro studies that have established a possible pathway to understand how DDT impacts female infertility and leads to reproductive cancers, along with epidemiologic studies that specifically targeted exposure to DDT of females belonging to various ethnicities to develop an overall picture of prevailing reproductive health concerns in different nations.

## 2. Methodology

Medline (Ovid Medline Epub Ahead of Print, In-Process & Other Non-Indexed Citations, Ovid MEDLINE(R) Daily, and Ovid MEDLINE(R)) and Google Scholar were systematically searched to detect all relevant animal and human studies published during the last 20 years (January 2003 to February 2023) in accordance with the Preferred Reporting Items for Systematic Reviews and Meta-Analyses (PRISMA) statement [19] using the following search terms “DDT”, “DDT and Female infertility” and “DDT and female reproductive tract cancers”, “Cervical Cancers”, “Ovarian and Uterine Cancers”. We primarily focused on research articles containing epidemiological information on human and animal data, including mechanistic and molecular information. Reference lists of selected studies were screened for other potentially eligible studies. After excluding duplicates, citations in abstract form, and non-English citations, the titles/abstracts of full papers were screened for relevance, defined as original research focusing on the topic “Effects of DDT AND Female infertility AND Female reproductive tract cancers”.

**Figure 1:**
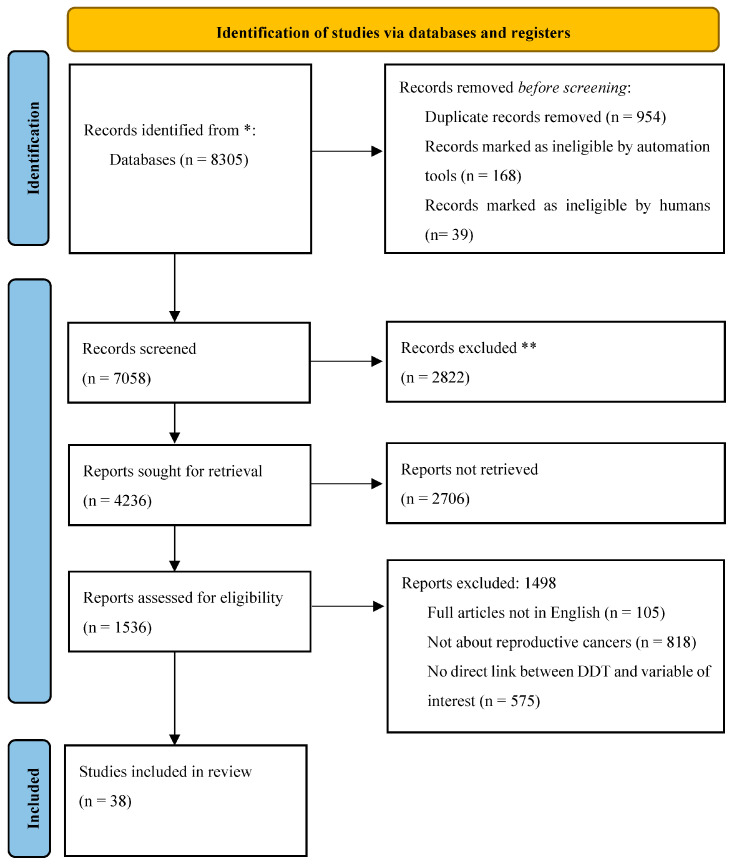
PRISMA flowchart. * Consider, if feasible to do so, reporting the number of records identified from each database or register searched (rather than the total number across all databases/registers). ** If automation tools were used, indicate how many records were excluded by a human and how many were excluded by automation tools.

In this systematic search and review, a total of 38 studies were finally included for qualitative synthesis (20–57). Figure 1 provides a visualization of the review process.

## 3. Role of DDT in Female Infertility

### 3.1. In Vitro Studies

Experimental studies investigating the role of DDT and its metabolites in female infertility include in vitro assessments on follicular [20], granulosa, endometrial [21], chorion [22], and placental cell cultures [23]. Six of the twelve in vitro assays included in this systematic search and review were conducted on cows [21,22,23,24,25,26], two on pigs [20,27], one on hamsters [28], two on Sprague–Dawley (SD) rats [29,30], and one on *Danio rerio* [31]. DDT and its metabolites impair fertility via hormone interference, cell cytotoxicity, and inhibition of enzymatic activity disrupting the ion balance, membrane permeability, uterine contractility, and ovarian steroidogenesis. This ultimately increases the risks of spontaneous abortions, miscarriages, delayed puberty, and reduced fecundability. Traces of DDT and its metabolites, particularly *p*,*p*′-DDE, have been detected in the ovarian follicular cells of cattle, sheep, and pigs. The follicular cell membrane is permeable to low- and high-molecular-weight compounds. DDT and its metabolites tend to alter the structure and function of the follicular cell membrane and eventually the oocyte membrane, which can have detrimental impacts on oocyte maturation, development of granulosa cells, and hormone secretion, which lead to impaired fertility over time.

**Figure 2:**
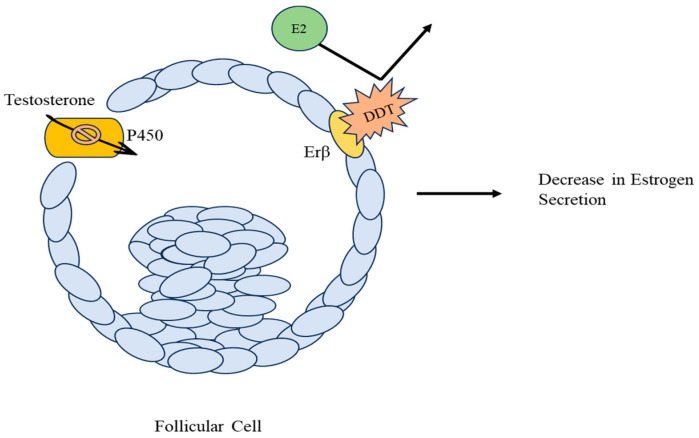
Reduction of estrogen in follicular cells. DDT acts as an antagonistic ligand to the ERβ receptor of E2 if exposed repeatedly and inhibits the enzymatic activity of Cytochrome P450, both of which result in reduced estrogen secretions. These secretions are altered through inhibitory pathways that reduce the breakdown of estrogen and formation of inactive E2 sulfates as a result of single exposure.

The mechanisms via which DDT and its metabolites cause fluctuations in the normal processes and maintain the balance of the reproductive system and associated hormones are complex and vary among different studies. Estradiol (E2) is produced in the ovaries and is responsible for fertility in non-pregnant women of childbearing age. DDT has both estrogenic and anti-estrogenic properties in a dose-and-exposure-dependent manner. At lower doses, *ο*,*p*′-DDT, *ο*,*p*′-DDE, and *ο*,*p*′-DDD decrease E2 secretion, indicating their anti-estrogenic nature, while at higher doses, they exhibit estrogenic characteristics. On the other hand, *p*,*p*′-DDT and *p*,*p*′-DDE are estrogenic independent of dosages but their repeated exposure decreases E2 levels [20]. To confirm the disruptive action of DDT, authors later studied its impact on ovarian steroidogenesis. They established DDT as an Estrogen Receptor Beta (Erβ) agonist and antagonist, corroborating their earlier findings that DDT is both estrogenic and anti-estrogenic [31]. However, no sole mechanism can be considered a standard pathway via which DDT decreases E2 secretion. DDT has anti-androgenic properties, particularly *p*,*p*′-DDE [31]; thus, it reduces testosterone levels, the prime substrate for Cytochrome P450 Enzymes. These P450 enzymes are responsible for the oxidation of xenobiotics, such as DDT, and for converting androgens into estrogens. Thus, DDT either reduces E2 synthesis via antagonistic action on the Erβ receptor, reducing the testosterone levels, which leads to less conversion of androgens into estrogens and inhibits the P450 activity via a lack of substrate [27], which can be visualized in Figure 2.

**Figure 3:**
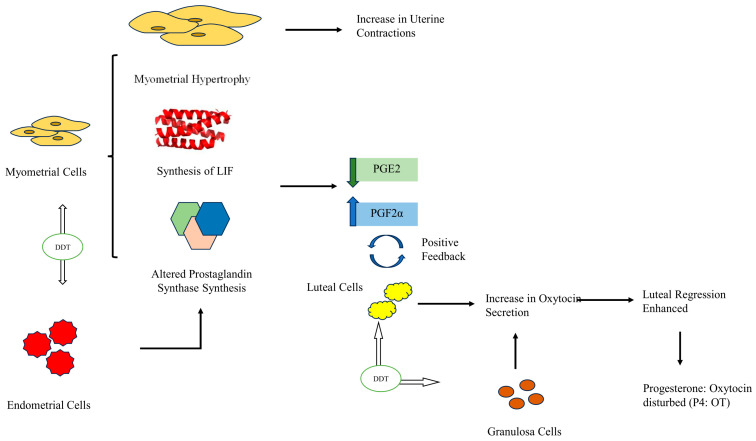
Effect of DDT on myometrial, endometrial, granulosa, and luteal Cells. DDT causes myometrial hypertrophy, which increases uterine contractions and increases the synthesis of Leukemia Inhibitory Factor (LIF) and alters the synthesis of prostaglandins in myometrial and endometrial cells, resulting in reduced PGE2 levels and elevated PGF2∝ levels. The increase in PGF2∝ is in a positive feedback mechanism with the secretion of oxytocin by luteal cells. Along with luteal cells, granulosa cells also secrete more oxytocin under the impact of DDT, which enhances the luteal regression, thereby disrupting the P4: OT. Altered prostaglandin synthesis and increased luteal regression disturb the overall P4: OT and muscular hypertrophy in the myometrium causes an increase in uterine contractions. The black arrows indicate the path while the broader white arrows indicate that DDT impacts on the mentioned cells.

**Figure 4:**
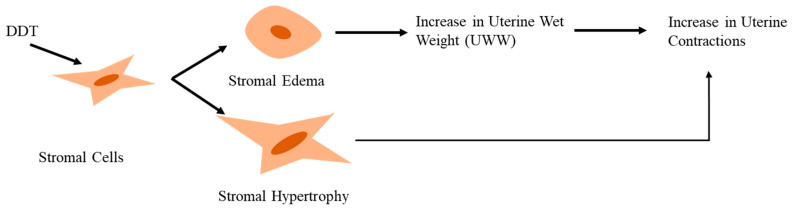
DDT-exposed stromal cells. DDT causes stromal hypertrophy and edema which result in increased uterine wet weight (UWW), both of which increase uterine contractions.

Lyche et al. [31] reported delayed puberty, skewed sex ratio, and feminization during pubertal development as possible implications of androgen inhibition by DDT and its metabolites. DDT and DDE increase oxytocin (OT) secretion from granulosa and luteal cells [21]. This finding is further supported by Mlynarczuk et al. [24] through transcriptome analysis, which displayed increased mRNA expression for Neurophysin-1 (NP-I)/oxytocin (OT) in pregnant cows, particularly at 9–12 weeks. During the same pregnancy duration, the mRNA expression for Prostaglandin A (PGA) is also observed to decrease in luteal cells, thus indicating the impact of DDT and its metabolites on prostaglandin synthesis. In mammals, Prostaglandin F2 alpha (PGF2α) and Prostaglandin E2 (PGE2) are important for the establishment of early pregnancy and for the course of the estrous cycle. Any imbalances in their ratios can have serious implications, particularly for fertilization and embryo implantation. Wrobel et al. [21] indicated that DDT and DDE influence the endometrial cells to secrete more PGF2a and less PGE2. This PGF2a:PGE2 ratio can increase myometrial contractions and accelerate luteal regression, which may alter progesterone (P4) levels responsible for pregnancy maintenance. A positive feedback loop exists between PGF2a and OT secretion during luteal regression. Thus, the increase in PGF2a is responsible for accelerated luteal regression and OT secretion. While performing a study on chorion explants obtained from cows, Mlynarczuk et al. [22] reported similar results by indicating that DDT impairs the secretion of PGE2, PGF2α OT, and P4 from the smooth chorion. A study conducted on pregnant cows indicated that only DDE increases P4 levels. However, the ratio of P4 to OT decreases in response to DDT and DDE. In the case of pregnant cows, this alteration in secretion patterns can induce strong myometrial contractions of the uterine strips [24]. This disturbs the regulation of processes in the cattle placenta and increases the risk of abortions and preterm/premature births [22], which has also been proven by multiple epidemiologic studies on human female subjects. Kwekwl et al. [29] also reported uterine motility dysregulation in mice and rats by *ο*,*p*′-DDT via species-specific uterine hypertrophy. Myometrial hypertrophy was more evident in mice that increased their uterine wet weight (UWW), while stromal hypertrophy was observed in rats. Stromal edema was, however, common in both species. In addition, Luminal Epithelial Height (LEH), a characteristic marker for estrogen exposure in the uterus, is also increased under the influence of *ο*,*p*′-DDT, indicating that the chemical is somewhat estrogenic in nature. A comprehensive depiction of these interlinked processes is shown in Figure 3 and Figure 4.

**Figure 5:**
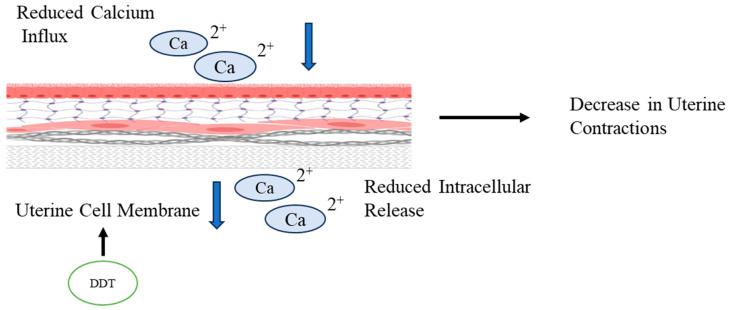
Ion imbalance in the uterine cell membrane. DDT causes reduced influx and intracellular release of calcium Ions through the uterine cell membrane, which results in decreased uterine contractions. The blue arrows indicate reduction in influx and release of Calcium Ions and the black ones indicate the effect and exposure of DDT.

On the contrary, Salleh et al. [30] showed that exposure to DDT reduces uterine contractions in rats. He proposed two mechanisms via which DDT impacts uterine motility and contractions: through inhibition of uterotonic pathways such as PGF2a and OT secretion, which have previously been mentioned; or through alteration in the Ca^2+^ influx and intracellular release, as shown in Figure 5, which ultimately reduces the uterine contractions. These compounds may also stimulate P4 secretion independently of E2 secretion from the chorion, which increases the ratio of P4 to E2 [25]. The levels of E2 and P4 maintain the placental barrier and maternal–fetal connections by regulation of the connexin (Cx) genes Cx26 and Cx43, the expressions of which are observed to decrease and increase, respectively, under the influence of DDT. DDE exposure increased Cx32 and Cx43 expression in the placental site [23]. These chemical and hormonal changes are speculated to impair the placental barrier function, disrupt trophoblast invasion, and impact the secretory activity of the placenta in cows [25]. Uterine contractions are important in transporting sperm, ovum, and placental and fetal expulsion at birth. Any dysregulation might lead to various adverse effects on fertility and reproduction.

**Figure 6:**
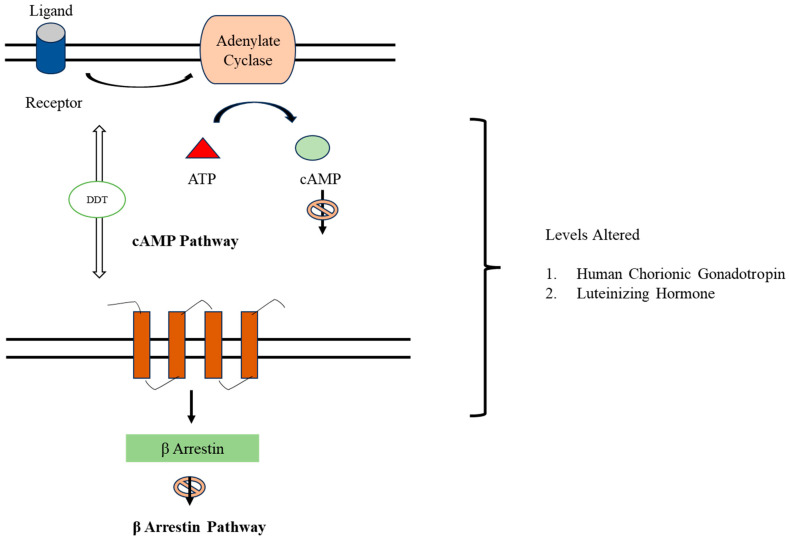
Pathway Inhibition by DDT. DDT alters the levels of human chorionic gonadotropin (hCG) and Luteinizing Hormone (LH) by inhibition of cAMP and β-arrestin pathways.

*p*,*p*′-DDT decreases the activity of human chorionic gonadotropin/Luteinizing Hormone Receptor (hCG/LHR) in a dose-dependent manner via negative allosteric modulation of the beta-arrestin 2 (B-arrestin 2) and cyclic 3′,5′-monophosphate (cAMP) pathways. These alter the levels of human chorionic gonadotropin (hCG), which is required to support pregnancy, and Luteinizing Hormone (LH), which is crucial to preparation of the uterus for pregnancy, leading to an increased risk of spontaneous abortions and miscarriages [28] (Figure 6).

The viability of the cells was unaffected by the doses of DDT and DDE used by Wrobel et al. [21] in their study. Thus, these alterations in the secretions of PGF2a, PGE2, and OT by endometrial and granulosa cells cannot be attributed to their cytotoxic effects. Instead, it could be that DDT and DDE impair prostaglandin synthesis directly by impacting the synthesis of a common precursor or specific synthases, or both, or indirectly by stimulating the Leukemia Inhibitor Factor (LIF) synthesis in the myometrium. LIF regulates decidualization and embryo–endometrial interaction, and makes the uterus receptive to implantation [26]. The dysregulation of hormone levels in response to DDT and its metabolites is summarized in Table 1 [20,21,22,24,25,27,28,31].

### 3.2. Epidemiological Studies

The epidemiological studies included in the systematic search and review consist of six case–control studies [32,33,34,35,36,37], seven cohort studies [38,39,40,41,42,43,44], two cross-sectional studies [45,46], and three pilot studies [47,48,49], which were conducted on women from different geographical areas with different ethnic backgrounds. The ethnicities of the women involved in the studies include American, Mexican, Indian, Chinese, Bolivian, Latina, German, and Laotian. The results of this systematic search and review reveal that out of 18 epidemiological studies included in the meta-analysis, 13 (72.2%) showed a negative association between DDT and female infertility, while 1 study (5.5%) showed a positive association between DDT and female fecundability, revealing increasing rates of conception with increased exposure. In contrast, four studies (22.2%) showed no association between DDT and female reproductive health. This suggests that many of the studies included show consistent results. DDT and its metabolites exert adverse effects on female fertility and reproductive health through various mechanisms of action, either by their activity as endocrine disruptors, anti-androgens, and xenoestrogens, or through interference in the binding of progesterone to its receptor, thereby disrupting the closure of sodium ion channels in membranes, as well as causing oxidative stress by the formation of reactive oxygen species (ROS). These detrimental effects of exposure to DDT and its metabolites can cause infertility, reduced fecundability, preterm birth (PTB), fetal loss, shorter time-to-pregnancy (TTP), decreased length of gestation (LOG), and reduced menstrual cycle length. An overview of how DDT and its metabolites impacted women belonging to different ethnicities is presented in Table 2 [32,33,34,35,36,37,38,39,40,41,42,43,44,45,46,47,48,49].

#### 3.2.1. DDT and Menstrual Cycle Length

It has been reported by several studies that DDT can reduce the length of the menstrual cycle in young women. Ouyang et al. [46] studied the effects of DDT exposure on age at first menstruation and menstrual cycle length. It was observed that a 10 ng/g increase in serum concentration of DDT reduced the age at which the first menstruation was experienced. The cause of this effect can be attributed to the activity of *p*,*p*′-DDE as an endocrine disrupter, which plays a role in increasing the amount of estradiol in the body by converting androgens to estrogens, in turn accelerating the maturation of oocytes causing early menarche. It was observed in the study that *p*,*p*′-DDE can increase the activity of human granulosa cells in combination with FSH. Therefore, it can increase the amount of estradiol in the body due to the action of granulosa cells influenced by FSH, which can aromatize androgens to estrogens. Ouyang et al. hypothesized that this phenomenon can lead to an upset in the hormonal balance, implicating changes in the menstrual cycle. These findings can also be further confirmed by another study by Windham et al. [49], who researched the effects of exposure to organochlorides and their ramifications for female ovarian function. Women with a higher serum concentration of DDE had a reduction in their menstrual cycle length, as well as their luteal phase length and their progesterone metabolite levels. DDT has been reported to interact with the progesterone-response pathway in various ways, including binding to the progesterone receptor, inhibiting progesterone-induced enzymes, and decreasing the frequency of implantation of eggs, thereby causing sterility and decreased serum progesterone levels, as observed in rats exposed to high levels of DDT. In this way, it can also alter the luteal phase length, making it shorter due to decreased progesterone production. Although these studies prove the negative impact of DDT on menstrual cycles, Chen et al. [47] found that neither *p*,*p*′-DDT nor *o*,*p*′-DDT had any connection with menstrual cycle length, duration of menses, or heaviness of menstrual flow in a pilot study they conducted on 60 Chinese women. This confirms that more investigation is required to thoroughly understand the mechanism behind the influence of DDT on the menstrual cycle.

#### 3.2.2. DDT and Infertility/Fecundability

Several epidemiologic and exposure studies in the scientific literature, some of which are discussed below, have provided evidence that DDT at low levels of exposure can cause adverse reproductive outcomes in humans and animals. However, many of the studies analyzing the relationship between DDT and infertility focus on males, while the number of female reports is meager. According to the presently available data on DDT and its impact on female reproductive health, DDT has an inverse relationship with female fertility. Cohn et al. [34] assessed the fecundability ratios for daughters exposed to maternal DDT and its metabolites in utero to monitor any induced transgenerational effects. According to the results *p*,*p*′-DDT decreased the daughters’ conception probability by 32% for every 10 μg/L *p*,*p*′-DDT present in maternal serum. Surprisingly, the opposite effect was observed with *p*,*p*′-DDE, as it increased the conception probability of daughters by 16% for every 10 μg/L *p*,*p*′-DDE in the maternal serum. The antiandrogenic effects of *p*,*p*′-DDT induce a detrimental impact on the ovaries during gestation and early life. However, the mechanism behind the positive effects of *p*,*p*′-DDE on conception is not well understood. In a similar study, Weiss et al. [48] found that German women with high concentrations of total serum DDT suffered from a lower pregnancy rate attributed to DDT’s xenoestrogenic behavior. Perry et al. [43] studied serum DDT levels and progesterone and estrogen levels across the menstrual cycle in women of reproductive age. The findings showed that DDT could cause a reduction in levels of estrogen during ovulation and of progesterone when it is required, as an indicator of corpus luteal function essential for early pregnancy maintenance. This can be attributed to the tendency of DDT to act as a xenoestrogen and an anti-androgen. Furthermore, DDT-PdG and E_1_C associations can harm female reproductive health, such as via impaired fertility and early pregnancy loss. The causal pathways of these associations are currently unknown but potentially involve multiple mechanisms including modifications in the biological synthesis and metabolism of sex steroids, along with receptor-mediated effects. DDT affects progesterone and estrogen metabolite profiles via multiple mechanisms, such as *p*,*p*′-DDE binding to the progesterone receptor or *o*,*p*′-DDT, and *o*,*p*′-DDE binding to the estrogen receptor, inhibiting the binding of endogenous estradiol. Kezios et al. [40] concluded that *p*,*p*′-DDE was negatively associated with the gestation length. It was also observed that *o*,*p*′-DDT and *p*,*p*′-DDE were linked with decreased birth weight, while the opposite was the case for *p*,*p*′-DDT. This could be because they operate through different biological pathways, leading to contrasting effects. While *p*,*p*′-DDE acts as an anti-androgen, *o*,*p*′-DDT is weakly estrogenic, thus causing the change in influence. Arrebola et al. [38] targeted the relationship between maternal *o*,*p*′-DDT and *p*,*p*′-DDE serum levels with birth outcomes. It was observed that *p*,*p*′-DDE was positively associated with birth weight, while the opposite was the case for *o*,*p*′-DDT. Ouyang et al. [42] reported that the women who were deficient in vitamin B2 but had high DDT serum concentrations were more susceptible to reduced clinical pregnancy (CP) and early pregnancy loss (EPL). In cases where Vit B was sufficient, DDT was not linked with CP, suggesting Vit B may help protect women against the adverse reproductive effects of DDT exposure. Although most studies found a negative association between DDT exposure and female fertility, Harley et al. [45] determined that there was no association between *p*,*p*′-DDT, *o*,*p*′-DDT, or *p*,*p*′-DDE and TTP in a cross-sectional study performed on 402 Latina women. This indicates insufficient research regarding women’s reproductive health and that this subject needs to be studied further.

#### 3.2.3. DDT and Preterm Birth

DDT has been associated with preterm birth (PTB) and reduced LOG and TTP. Torres-Arreola et al. [36] conducted a case–control study to determine the association between maternal serum *p*,*p*′-DDE levels and preterm birth. It was observed that *p*,*p*′-DDE increased the risk of preterm birth by twofold at low levels (OR = 1.87, 95% CI = 0.95–3.68 for 111.6–228.8 ng/g and OR = 1.67, 95% CI = 0.84–3.31 for >228.8 ng/g). It was observed that *p*,*p*′-DDE exhibited a dose–response effect on preterm birth at levels exceeding 21 μg/L. The exact process explaining the correlation between *p*,*p*′-DDE, and preterm birth is not yet determined. However, the antiandrogenic behavior of *p*,*p*′-DDE and its interference in the binding of progesterone to its receptor could be attributed to its effect on pregnancy duration, consequently causing preterm birth. Tyagi et al. [37] studied the accumulation of POPs in pregnant women and the placenta to examine the possible effects of their exposure on PTB and LOG. Women with substantial levels of DDT made up 8.8% of PTB cases, contrasting with 2.7% for mothers with no detectable levels of DDT in their blood. A significant amount of DDT was also found in the placental tissues of PTB cases. A negative correlation was also found between LOG and DDE levels in maternal blood and high levels of DDT in breast milk, suggesting it has a role in early birth. Anand et al. [32] analyzed the residues of OCP in the placenta of females in preterm and full-term deliveries to evaluate the levels of different oxidative stress markers and to correlate them with OCP levels. The investigation revealed that oxidative stress caused by OCP residues plays a significant role in preterm deliveries. It was hypothesized that oxidative stress in trophoblastic placental tissue caused by the generation of reactive oxygen species (ROS) resulted in adverse reproductive outcomes. However, it could also be plausible to assume that the xenoestrogenic nature attributed to DDT alters the natural hormonal balance between estrogen and progesterone responsible for maintaining pregnancy, causing harmful pregnancy outcomes. Anand et al. [33] conducted another study on 90 Indian women and observed that women exposed to OCP were 1.7 times more likely to deliver a preterm baby than pregnant women who were not exposed. Around 100% of pregnant women had detectable levels of *p*,*p*′-DDE in their placenta and umbilical cord in PTB cases, confirming that OCP compounds are responsible for reproductive toxicity. Farhang et al. [39] examined 20,754 women in a longitudinal cohort study to study the effects of maternal serum DDT and DDE concentrations and their influence on male infants’ PTB, SGA, and birth weight. The results showed that no statistically significant relationship existed between the serum measurements of DDT or DDE and birth weight, length of gestation, and SGA. However, these results could not be generalized to female infants.

#### 3.2.4. DDT and Fetal Loss

The endocrine disruptive behavior of DDT allows it to inhibit the deactivation and inactivation of the sodium channels in the membrane of tissues such as the placenta, causing prolonged currents that keep the channel from closing. Longnecker et al. [41] verified this in their study, which aimed to confirm the relationship between maternal serum DDE levels and fetal loss in previous pregnancies. The results revealed that increasing maternal serum DDE levels were positively associated with fetal loss, while DDT showed no interconnection. The odds ratio was 1.4 (95% CI 1.1–1.6) for a 60 μg/L increase in serum level. The mechanism of action was postulated to be acute DDT toxicity induced by obstruction or closure of sodium channels in the placenta. The antiandrogenic behavior of DDE, blocking progesterone from binding to its receptor, was also considered to be a valid mechanism of action in causing fetal loss. Venners et al. [44] carried out a similar study with the aim of determining how preconception serum total DDT had an impact on pregnancy loss. Among the DDT metabolites, the most abundant in concentration was *p*,*p*′-DDE, which made up around 92%, while *p*,*p*′-DDT accounted for 6% of the total mass of DDT metabolites. It was estimated that every 10 ng/g (OR = 1.19, 95% CI 1.04, 1.36) increase in serum total DDT was linked to the relative odds of total and early pregnancy losses. Results confirmed the positive linear correlation of serum total DDT concentration and early pregnancy losses. Although the causal pathway could not be identified, it was hypothesized that the disruption in the closure of sodium channels caused by DDT and its tendency to prevent progesterone from binding to its receptor could be relevant to the cause of early fetal loss. Mahalingaiah et al. [35] demonstrated the link between DDT and DDE serum levels with implantation failure, chemical pregnancy, and spontaneous abortion in women undergoing in vitro fertilization procedures. The study concluded that no statistically significant associations existed between serum and follicular concentrations of DDT/DDE and oocyte number, quality, fertilization, or pregnancy rates. It was determined that the likelihood of implantation failure was increased due to the suppression of luteal progesterone, leading to the incomplete maturation of the endometrial lining or early pregnancy loss due to significantly low levels of progesterone production. The effects of exposure to DDT on female infertility and its mode of action are not entirely understood with the current information available. Further investigations and studies are required to determine the full extent of the potentially harmful impacts of exposure to DDT on females’ reproductive health.

## 4. Role of DDT in Female Reproductive Cancers

Cervical, ovarian, and uterine cancers are prevalent cancers among women from all regions and DDT has been found in their samples. However, during the past 20 years, few studies have been conducted regarding the impact of DDT on female reproductive tract cancers. The eight studies regarding the role of DDT in female reproductive cancers included in this review mainly highlight the presence of DDT and its metabolites in female reproductive cancer patients [50,51,52,53,54,55,56,57].

Ndebele et al. [54] conducted a study to understand the mechanism through which xenoestrogens such as DDT modulate cervical cancer biology. An increase in Cyclin A and D protein levels was observed to variable degrees, indicating that DDT induces changes in B-cell lymphoma 2 (BCL-2), the protein that regulates cell death. It also suppressed HeLa cell proliferation in a concentration-dependent manner and, thus, has cytotoxic effects on cells. Earlier findings also reported the influence of DDT on the development and growth of leiomyoma by affecting angiogenesis and apoptosis [50]. According to Mathur et al. [53], although women aged 41–50 had a higher risk of developing reproductive tract cancers, higher DDT residues were more prevalent in women aged 21–30 in Jaipur, India. Gibson and Saunders [51] worked on 524 Ishikawa cell lines established from endometrial adenocarcinoma cells of a 39-year-old woman, and observed that specifically *o*,*p*′-DDT had 100-fold more estrogenicity in reproductive tissues than *p*,*p*′-DDT. A case–control study conducted in North India also reported significant results regarding the presence of DDT and DDE at higher levels in the blood among epithelial ovarian cancer patients [57]. Kalinina et al. [52] treated normal human endometrial cells with DDT and observed upregulation of miRNA 190b. A similar pattern was reported in the uterus of rats, indicating an increase in the expression of oncogene Tumor protein p53-inducible nuclear protein 1 (*Tp53inp1*), which increases the chances of uterine cancers. The blood and cervical tissue samples from cervical cancer patients were studied to detect the levels of OCP among women of East Delhi. DDT was significantly higher in the blood samples of cases, while no correlation was observed between levels of DDT in cervical tissue samples and blood samples. The higher pesticide levels in the blood were also attributed to the region’s higher DDT content in wheat [55]. A similar study conducted in Mexico also showed higher DDE and DDD levels in blood samples. Women from Tizimin, Progresso, and Kanasin had high DDD, DDT, DDE, and DDD levels, respectively [56].

## 5. Conclusions

This systematic search and review indicated that exposure to DDT is associated with female reproductive health decline and that DDT acts as a carcinogen sufficient to instigate reproductive cancers. DDT causes the disruption of hormones that are primarily responsible for fertility and the maintenance of pregnancy, such as estrogen, progesterone, and oxytocin. This systematic review summarizes the various studies conducted over the last two decades to understand the underlying mechanisms of DDT action. The aim was to present a link between all of these studies, and thereby to develop an overall mechanism to establish a comprehensive understanding of the impact of DDTs on the female reproductive system. This is followed by an epidemiologic analysis, which serves to describe the prevailing reproductive issues in different parts of the world. However, due to the limited literature present on the association of DDT with female reproductive health and cancers, this study is limited to possible pathways of DDT that might result in female infertility and reproductive tract cancers, instead of establishing highly conclusive data. Thus, more research is still required to obtain substantial data to prove whether a strong link exists between all the pathways highlighted in this review.

## Figures and Tables

**Table 1:** Impact of DDT and its metabolites on hormone levels. DDT and its metabolites alter hormone secretion levels by increasing or decreasing their production through various mechanisms.

Chemical/Hormone	DDT Isomer	Levels of Chemical/Hormone
Estradiol	*ο*,*p*′-DDT	Increase or Decrease
	*ο*,*p*′-DDE	Increase or Decrease
	*ο*,*p*′-DDD	Increase or Decrease
	*p*,*p*′-DDT	Increase
	*p*,*p*′-DDE	Increase
Oxytocin	DDT	Increase
	DDE	Increase
Prostaglandin A	DDT	Decrease
	DDE	Decrease
Prostaglandin F2a	DDT	Increase
	DDE	Increase
Prostaglandin E	DDT	Decrease
	DDE	Decrease
Progesterone	DDE	Increase
hCG/LHR	*p*,*p*′-DDT	Decrease

**Table 2:** Impact of DDT and its metabolites on reproductive disorders based on ethnicity. DDT and its metabolites were associated with the incidence of various parameters of reproductive disorders in women having different ethnic backgrounds.

Sr No.	Study Type	Ethnicity	Population	Metabolite	Parameter	Effect
1	Case–control	American	289	*p*,*p*′-DDT, *p*,*p*′-DDE	TTP	Decreased
2	Case–control	Mexican	233	*p*,*p*′-DDE	PTB	Increased
3	Nested case–control	American	720	DDT/DDE	Infertility	No statistically significant association
4	Case–control	North Indian	100	DDT/DDE	PTB	Increased
5	Case–control	Indian	90	*p*,*p*′-DDE	PTB	Increased
6	Case–control	Indian	90	*p*,*p*′-DDT, *p*,*p*′-DDE	PTB	Increased
7	Cohort	American	2613	DDT/DDE	Fetal loss	DDE increased, no relation with DDT
8	Cohort	American	20,754	DDT/DDE	PTB	No statistically significant association
9	Cohort	Chinese	287	*o*,*p*′-DDT, *o*,*p*′-DDE	Menstrual cycle length	Increased
10	Cohort	American	1752	*p*,*p*′-DDT, *o*,*p*′-DDT, *p*,*p*′-DDE	POG	Decreased
11	Prospective	Chinese	291	DDT	Clinical pregnancy	Decreased
12	Cohort	Bolivian	200	*p*,*p*′-DDE, *o*,*p*′-DDT	POG	Decreased
13	Prospective	Chinese	388	DDT	Fetal loss	Increased
14	Cross-sectional	Chinese	466	*p*,*p*′-DDE/DDT	Menstrual cycle length	Reduced age at menarche
15	Cross-sectional	Latina	402	*p*,*p*′-DDT, *o*,*p*′-DDT, *p*,*p*′-DDE	TTP	No statistically significant association
16	Pilot	Chinese	60	*p*,*p*′-DDT, *o*,*p*′-DDT	Menstrual cycle length	No statistically significant association
17	Pilot	German	89	DDT	Infertility	Increased
18	Pilot	Laotian	50	DDT/DDE	Menstrual cycle length	Decreased

## Abbreviations

AR	Androgen Receptor
cAMP	Cyclic Adenosine 3′, 5′-monophosphate
CP	Clinical Pregnancy
Cx	Connexin
DDD	Dichlorodiphenyldichloroethane
DDE	Dichlorodiphenyldichloroethylene
DDT	Dichlorodiphenyltrichloroethane
E2	Estradiol
EDC	Endocrine-disrupting Chemicals
EPL	Early Pregnancy Loss
ER	Estrogen Receptor
Erβ	Estrogen Receptor Beta
FSH	Follicle Stimulating Hormone
hCG	Human chorionic gonadotropin
hCG/LHR	Human chorionic gonadotropin/Luteinizing Hormone Receptor
LRAT	Long-Range Atmospheric Transport
LEH	Luminal Epithelial Height
LH	Luteinizing Hormone
LIF	Leukemia Inhibitor Factor
NP-I	Neurophysin-1
*ο*,*p*′-DDD	ortho, para′-Dichlorodiphenyldichloroethane
*ο*,*p*′-DDE	ortho, para′-Dichlorodiphenyldichloroethylene
*ο*,*p*′-DDT	ortho, para′-Dichlorodiphenyltrichloroethane
OCP	Organochlorine Pesticide
OCP	Organochlorine Pesticide
OT	Oxytocin
*p*,*p*′-DDE	para, para′-Dichlorodiphenyldichloroethylene
*p*,*p*′-DDT	para, para′-Dichlorodiphenyltrichloroethane
*p*,*p*′-DDD	para, para′-Dichlorodiphenyldichloroethane
P4	Progesterone
PGA	Prostaglandin A
PGE2	Prostaglandin E2
PGF2α	Prostaglandin F2 alpha
LOG	Length of gestation
POP	Persistent Organic Pollutant
PRISMA	Preferred Reporting Items for Systematic Reviews and Meta-Analyses
PTB	Preterm Birth
ROS	Reactive Oxygen Species
SGA	Small for Gestational Age
TTP	Time to Pregnancy
UWW	Uterine Wet Weight
